# Heart-Ankle Pulse Wave Velocity Is Superior to Brachial-Ankle Pulse Wave Velocity in Detecting Aldosterone-Induced Arterial Stiffness

**DOI:** 10.3390/biomedicines9101285

**Published:** 2021-09-22

**Authors:** Zheng-Wei Chen, Chien-Ting Pan, Cheng-Hsuan Tsai, Yi-Yao Chang, Chin-Chen Chang, Bo-Ching Lee, Yu-Wei Chiu, Wei-Chieh Huang, Yu-Li Lin, Vin-Cent Wu, Chi-Sheng Hung, Che-Wei Liao, Yen-Hung Lin, on behalf of TAIPAI Study Group

**Affiliations:** 1 Department of Internal Medicine, National Taiwan University Hospital and National Taiwan University College of Medicine, Taipei 100, Taiwan; librajohn7@hotmail.com (Z.-W.C.); pan.chienting.m@gmail.com (C.-T.P.); cheng.hsuan.richard.tsai@gmail.com (C.-H.T.); dr.vincentwu@gmail.com (V.-C.W.); petrehcs@gmail.com (C.-S.H.); 2 Department of Internal Medicine, National Taiwan University Hospital Yun-Lin Branch, Yun-Lin 640, Taiwan; 3 Department of Internal Medicine, National Taiwan University Hospital Jin-Shan Branch, New Taipei City 208, Taiwan; 4 Department of Cardiovascular Medicine, Far Eastern Memorial Hospital, New Taipei City 220, Taiwan; rollerpapa@gmail.com (Y.-Y.C.); dtmed005@yahoo.com.tw (Y.-W.C.); 5 Department of Medical Imaging, College of Medicine, National Taiwan University Hospital and National Taiwan University, Taipei 100, Taiwan; macotocc@gmail.com (C.-C.C.); bochinglee@gmail.com (B.-C.L.); 6 Department of Computer Science and Engineering, Yuan Ze University, Taoyuan City 320, Taiwan; 7 Division of Cardiology, Department of Internal Medicine, Taipei Veterans General Hospital, Taipei 112, Taiwan; hwcc0314@gmail.com; 8 Department of Business Administration and Graduate School of Service Management, Chihlee University of Technology, New Taipei City 220, Taiwan; elee@mail.chihlee.edu.tw; 9 Department of Medicine, National Taiwan University Cancer Center, Taipei 106, Taiwan

**Keywords:** primary aldosteronism, arterial stiffness, brachial-ankle pulse wave velocity, heart-ankle pulse wave velocity

## Abstract

Primary aldosteronism (PA) is associated with higher arterial stiffness compared to essential hypertension (EH). However, few studies have compared different pulse wave velocity (PWV) parameters to detect aldosterone-induced arterial stiffness. In this study, we aimed to compare the sensitivity in detecting aldosterone-induced arterial stiffness between brachial-ankle PWV (baPWV) and heart-ankle PWV (haPWV). We prospectively enrolled 1006 PA patients and 983 EH patients. Detailed medical history, basic biochemistry data and two PWV measurements (baPWV and haPWV) were collected in both groups. We performed analysis on the original cohort and two propensity score matching (PSM) models (model 1 adjusted for age and sex; model 2 adjusted for age, sex, systolic and diastolic blood pressure). The DeLong test was used to compare areas under receiver operating characteristic curves (AUCs) between baPWV and haPWV to predict PA. In all models, the PA patients had significantly higher baPWV compared to the EH patients. The AUC of haPWV was greater than that of baPWV. In conclusion, haPWV seems to be a better PWV parameter than baPWV in detecting aldosterone-induced arterial stiffness.

## 1. Introduction

Primary aldosteronism (PA) is the most common endocrine-related secondary hypertension [1,2], accounting for 5–15% of all hypertensive patients, and the rate is even higher in patients with resistant hypertension [3,4]. The excess aldosterone in PA causes both cardiovascular structural and functional deterioration [5,6,7,8] and increased cardiovascular and cerebrovascular complications, including coronary artery disease, myocardial infarction, stroke, transient ischemic attack as well as arrhythmia and heart failure [9,10,11,12,13,14,15]. Microscopically, the overproduction of aldosterone leads to vascular endothelial dysfunction, which further causes vascular inflammation, vascular remodeling and increased arterial wall stiffness [16].

Pulse wave velocity (PWV) is a widely used non-invasive tool to evaluate arterial wall stiffness [17]. PWV is calculated as the distance between two different points along the arterial tree divided by the pulse wave propagation time [18]. A higher PWV value indicates increased arterial stiffness, which in turn indicates an increased cardiovascular risk [19]. Different segments of PWV measurements represent various degrees of contributions from central elastic arteries and peripheral muscular arteries [20]. For example, carotid–femoral (cfPWV), heart-carotid (hcPWV), and heart-femoral (hfPWV) PWV represent central artery stiffness; heart-ankle (haPWV) and brachial-ankle (baPWV) PWV represent the combination of central and peripheral stiffness; and femoral-ankle (faPWV) PWV represents peripheral arterial stiffness.

In 2006, Strauch et al., were the first group to demonstrate that a higher PWV indicated higher arterial stiffness in PA patients compared with essential hypertension (EH) patients [21]. CfPWV was the most commonly used modality in early studies [21,22,23]. Subsequently, Rosa et al. demonstrated increased peripheral muscular arterial stiffness in PA patients using faPWV [24]. In our previous studies, we used both baPWV and haPWV to assess arterial wall stiffness [25,26,27,28,29]. Anatomically, haPWV is a direct combination of central aorta and peripheral vasculature, while baPWV is not a simple unidirectional pathway for arterial pulse waves, and some segments of central and peripheral arteries may be lost [20]. In addition, the fibroproliferative effect of aldosterone overproduction has been shown to alter both central and peripheral arterial stiffness [24]. Therefore, haPWV may be more responsive to the changes in vascular stiffness caused by aldosterone than baPWV. However, no previous study has compared baPWV with haPWV for arterial stiffness measurements in PA patients. Therefore, the aim of this study was to compare the sensitivity in detecting aldosterone-induced arterial stiffness between baPWV and haPWV.

## 2. Materials and Methods

### 2.1. Patients

**Figure 1:**
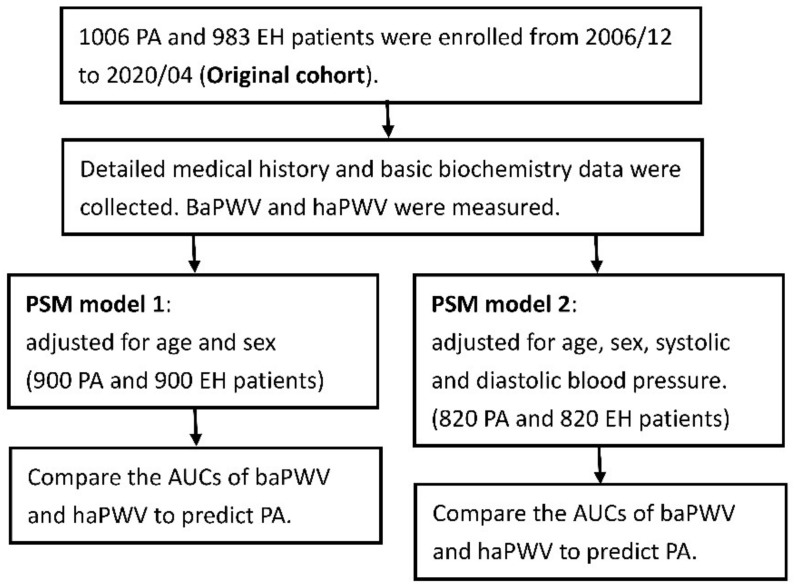
The designed patient flow diagram. Abbreviation: PA, primary aldosteronism; EH, essential hypertension; baPWV brachial-ankle pulse wave velocity; haPWV, heart-ankle pulse wave velocity; AUCs, areas under receiver operating characteristic curves.

We prospectively enrolled 1006 PA patients from December 2006 to April 2020. Another 983 EH patients were enrolled as the control group. The patient flow diagram was shown in Figure 1. All PA patients were registered in the Taiwan Primary Aldosteronism Investigation (TAIPAI) database [30]. All EH patients were enrolled during the study period. EH was diagnosed by exclusion after appropriate evaluation of all detectable forms of secondary hypertension according to standard algorithms. A comprehensive medical history was documented and basic biochemistry data were measured at the first assessment. Plasma aldosterone concentration (PAC) was measured using a commercial radio-immune assay kit (ALDO-RIACT RIA kit, Cisbio Bioassays, Codolet, France). Plasma renin activity (PRA) was measured as the generation of angiotensin-I in vitro using a commercially available radio-immune assay kit (GammaCoat, DiaSorin, Stillwater, MN, USA).

This study was conducted according to the Declaration of Helsinki, and it was approved by the Institutional Review Board (200611031R) of National Taiwan University Hospital (Taipei, Taiwan). Informed consent was obtained from all participants before enrollment.

### 2.2. Diagnostic Criteria for Primary Aldosteronism

The diagnosis of PA was made according to the following three criteria: autonomous excess aldosterone production with an aldosterone-to-renin ratio more than 35; a TAIPAI score more than 60% [31]; and post-saline loading PAC more than 10 ng/dL, or PAC/PRA more than 35 (ng/dL)/(ng/mL per h) in a post-captopril test, or PAC more than 6 ng/dL in a fludrocortisone suppression test [32]. The patients were asked to discontinue all antihypertensive medications for at least 21 days before PAC and PRA measurements. Diltiazem and/or doxazosin were administered to control markedly high blood pressure if needed.

### 2.3. PWV Measurements

PWV was acquired using an automatic waveform analyzer (Colin VP-2000, Omeron Inc., Japan) after 15 min of rest in a supine position [26]. The instrument simultaneously recorded the pressure waveforms of bilateral brachial and tibial arteries, phonocardiograms and electrocardiograms. Occlusive cuffs connected to oscillometric and plethysmographic sensors were wrapped around the upper arms and ankles for blood pressure and pulse waveform measurements and analysis. The distance between the arms and ankles was estimated according to the body height of the patient. The time taken for an arterial pressure wave to travel between brachial points and ankle points was calculated according to wave front theory. The baPWV was obtained from the ratio of arm-ankle distance to arm–ankle time difference, and the haPWV was obtained using the same protocol. We measured both right and left side PWV, and the mean values of baPWV and haPWV were calculated.

### 2.4. Statistical Analysis

Data were expressed as number (%) for categorical data, mean ± standard deviation for normally distributed variables, and median (25th–75th interquartile range) for non-normally distributed variables. All continuous variables were compared between the PA and EH patients using the Student’s t test if normally distributed, or the Wilcoxon rank-sum test if non-normally distributed. Differences between proportions were assessed using the chi-square test or Fisher’s exact test. A two-sided *p* value <0.05 was considered to be statistically significant. All statistical analyses were performed using SPSS version 25 for Windows (SPSS Inc., Chicago, IL, USA). The R-3.3 plugin extension was used to perform propensity score analysis. A non-parsimonious multiple logistic regression model with possible confounding factors, including age, sex and systolic and diastolic blood pressures was used to estimate propensity scores in the PA and EH patients. A 1:1 matching ratio was used with a caliper width equal to 0.03 standard deviation of the logit of the propensity scores. Covariate balance between the matched groups was investigated. Receiver operating characteristic (ROC) curve analysis was used to assess the ability of haPWV and baPWV to differentiate PA and EH patients. ROC curves were plotted using STATA version 16 software (StataCorp LP, College Station, TX, USA). Areas under the ROC curves (AUCs) measured the entire area underneath the ROC curve by integral calculus. The DeLong test was used to compare AUCs between haPWV and baPWV.

## 3. Results

### 3.1. Clinical Characteristics

#### 3.1.1. Overall Patients before Propensity Score Matching (PSM)

Before PSM, a total of 1006 PA patients and 983 EH patients were enrolled for analysis. The baseline clinical characteristics of both groups are shown in Table 1 (left). The PA group had a lower percentage of males and was older than the EH group. The body weight, body mass index and serum creatinine level were similar between the PA and EH patients. The PA patients had a lower body height, higher systolic and diastolic blood pressures, lower potassium level, were prescribed with more types of antihypertensive medications, and had a longer duration of hypertension compared to the EH patients. Regarding the types of hypertensive medications for blood pressure control, the PA patients had higher prescription rates of alpha-blockers, beta-blockers, calcium channel blockers, vasodilators, spironolactone and diuretics.

#### 3.1.2. Propensity Score Matching for Age and Sex (PSM Model 1)

After 1:1 PSM for age and sex, there were 900 patients in each group. The clinical characteristics of the matched PA and EH patients are shown in Table 1 (middle). There were no significant differences in sex and age between the PA and EH patients. In PSM model 1, the PA patients still had higher systolic and diastolic blood pressures, lower potassium level, more types of antihypertensive medications and longer hypertension duration compared to the EH patients. The types of hypertensive medications for blood pressure control were similar before PSM and in PSM model 1.

#### 3.1.3. Propensity Score Matching for Age, Sex, Systolic and Diastolic Blood Pressures (PSM Model 2)

We further added systolic and diastolic blood pressures to age and sex in PSM to eliminate their possible hemodynamic effects on PWV measurements. After 1:1 PSM for age, sex, systolic and diastolic blood pressures, there were 820 patients in each group. The clinical characteristics of the matched PA and EH patients are shown in Table 1 (right). In PSM model 2, no significant differences in sex, age, systolic and diastolic blood pressures were noted between the matched PA and EH patients. The matched PA patients still had a lower potassium level, more types of antihypertensive medications and longer hypertension duration compared to the EH patients. In addition, the PA patients had a lower prescription rate of angiotensin receptor blockers and higher prescription rates of alpha-blockers, beta-blockers, calcium channel blockers, vasodilators, spironolactone and diuretics.

### 3.2. PWV Data

#### 3.2.1. Original Overall Patients before PSM

Before PSM, the PA patients had significantly higher baPWV [1637 (1452–1868) vs. 1527 (1362–1756) cm/s, *p* < 0.001] and haPWV [1103 (1008–1218) vs. 1040 (945–1150) cm/s, *p* < 0.001] compared with the matched EH patients (Table 2, left).

#### 3.2.2. PSM Model 1

In PSM model 1, the matched PA patients still had significantly higher baPWV [1616 (1440–1844) vs. 1544 (1371–1776) cm/s, *p* < 0.001] and haPWV [1093 (1003–1208) vs. 1052 (957–1158) cm/s, *p* < 0.001] compared with the matched EH patients (Table 2, middle).

#### 3.2.3. PSM Model 2

In PSM model 2, which eliminated the influence of hemodynamics, the matched PA patients still had significantly higher baPWV [1602 (1434–1816) vs. 1574 (1394–1797) cm/s, *p* = 0.047] and haPWV [1089 (995–1198) vs. 1062 (974–1167) cm/s, *p* = 0.001] compared with the EH patients (Table 2, right).

### 3.3. Receiver Operating Characteristic (ROC) Curve Analysis

#### 3.3.1. Original Overall Patients before PSM

**Figure 2:**
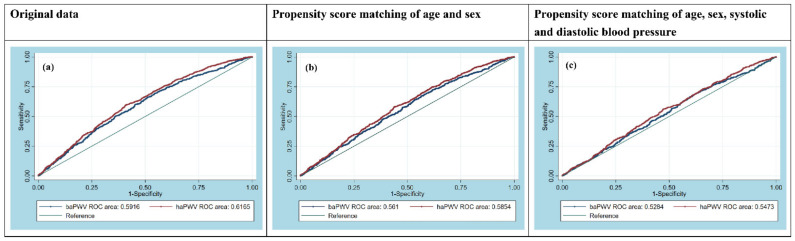
Receiver operating characteristic (ROC) curves for haPWV (red line) and baPWV (blue line) to differentiate PA and EH patients. (**a**) Original data; (**b**) Propensity score matching by age and sex (model 1); (**c**) Propensity score matching by age, sex, systolic and diastolic blood pressures (model 2). The areas under the ROC curves for haPWV and baPWV to differentiate PA and EH patients were 0.62 (95% confidence interval (CI) = 0.59–0.64) and 0.59 (95% CI = 0.57–0.62), respectively, in the patients overall, 0.59 (95% CI = 0.56–0.61) and 0.56 (95% CI = 0.53–0.59) in model 1 and 0.55 (95% CI = 0.52–0.58) and 0.53 (95% CI = 0.50–0.56) in model 2.

The ROC curves are illustrated in Figure 2, and the AUCs are summarized in Table 3. Before PSM, the AUC of haPWV was greater than that of baPWV (0.6165 [0.5920–0.6411] vs. 0.5916 [0.5667–0.6165], *p* = 0.0001) (Table 3, left; Figure 2a).

#### 3.3.2. PSM Model 1

In PSM model 1, the AUC of haPWV was still greater than that of baPWV (0.5854 [0.5592–0.6116] vs. 0.5610 [0.5346–0.5875], *p* = 0.0001) (Table 3, middle; Figure 2b).

#### 3.3.3. PSM Model 2

Similarly, the AUC of haPWV was still greater than that of baPWV in PSM model 2 (0.5473 [0.5195–0.5751] vs. 0.5284 [0.5004–0.5563], *p* = 0.0046) (Table 3, right; Figure 2c).

## 4. Discussion

Our results demonstrated that haPWV was more sensitive than baPWV to detect aldosterone-induced vascular damage independently of hemodynamics. Moreover, the superiority of haPWV still existed after PSM for age, sex, systolic, diastolic blood pressures and hypertension history (not shown in the results section). To the best of our knowledge, this study is the first to investigate and support the role of haPWV in arterial stiffness assessments in PA patients.

Previous studies have reported that the excess aldosterone in PA patients is associated with a higher incidence of cardiovascular events compared to EH patients independently of the effects of hypertension [9,10,11,12,13,14,15]. Regarding cardiac structure, PA patients have been shown to have higher percentages of left ventricular remodeling, left ventricle hypertrophy, and cardiac fibrosis, which lead to cardiac dysfunction in diastolic and even systolic components [8]. Regarding the vasculature, aldosterone-induced endothelial dysfunction has been shown to further impair vascular tone, inflammation response, vascular remodeling and early atherosclerosis [16]. Both cardiac and vascular changes are associated with increased clinical events. Increased arterial stiffness is a consequence of extracellular matrix remodeling, which occurs during aging and hypertension, and it has also been shown to progress more rapidly due to aldosterone overproduction in PA patients [33].

PWV is an index of arterial wall stiffness [17]. The segments of PWV can reveal the compositions of central elastic arteries and peripheral muscular arteries [20]. CfPWV is a traditional validated marker used to assess the arterial stiffness of central arteries, whereas baPWV and haPWV are increasingly being used due to the integration of both central and peripheral arteries. The inconstant mechanical properties along the arterial tree result in diverse responses to aging, hypertension, different diseases and status [34].

PA patients have been demonstrated to have increased central arterial stiffness as measured by cfPWV compared with EH patients [21,22,23]. Rosa et al. showed an increase in both central (cfPWV) and peripheral (faPWV) arterial stiffness in PA patients compared with EH patients, who were matched by age, blood pressure, body mass index, lipid profile, and fasting glucose [24]. Furthermore, they reported that aldosterone level in the PA patients was the only predictor of peripheral PWV, while age and systolic blood pressure were the main predictors for central PWV [24]. The authors concluded that the fibroproliferative effect of aldosterone was generalized without preference for central elastic artery [24]. Damiano et al. also reported increased total collagen and type III vascular collagen deposition, indicating more pronounced fibrosis, in small resistance arteries of PA patients than in blood pressure-matched EH patients [35]. However, higher serum and urine aldosterone levels have still been shown to predict higher central artery stiffness in EH patients [36]. Taken together, these studies emphasize the role of the renin–angiotensin–aldosterone system in peripheral muscular arteries.

In our previous studies, we used baPWV and haPWV, representing both central and peripheral vascular properties, to evaluate aldosterone-induced vascular damage [25,26,27,28,29]. The current study showed that haPWV was more sensitive than baPWV in identifying the vascular changes caused by aldosterone excess. One major reason is less variability in haPWV than baPWV measurements. Our previous studies demonstrated that the standard deviation of haPWV was smaller than that of baPWV when measured simultaneously [26,27]. In PSM model 2 in the current study, the haPWV and baPWV values in the PA patients were 1107 ± 160 and 1664 ± 349 cm/s (not shown in the results section). The lower standard deviation indicates that the obtained data were closer to the mean, which means that they could better predict information about the population based on the sample data [37]. Similarly, Kuo et al. also found that compared to baPWV, haPWV could more accurately identify the presence of moderate-to-severe white matter hyperintense abnormalities due to the lower discrepancy [38].

Anatomically, haPWV can be considered to be a direct combination of hfPWV (central) and faPWV (peripheral); whereas baPWV can be considered as the difference between haPWV and heart–brachial PWV. Thus, baPWV would lose partial segments of central arteries, from the heart to brachial areas. The higher value of baPWV than haPWV also indicated a higher proportion of peripheral muscular artery component in baPWV. Moreover, baPWV, as with cfPWV, is not a simple unidirectional pathway for arterial pulse waves, and the actual travelled distance is approximated by body height [39]. This may be why haPWV was more comprehensive than baPWV to detect vascular changes in PA patients. However, unlike the abundance of clinical evidence for baPWV [40,41], further studies are needed to validate the prognostic value of haPWV.

Although haPWV has not been extensively studied, the concept has been integrated into the cardio-ankle vascular index (CAVI), which is an arterial stiffness index proposed by Shirai et al. in Japan in 2006 [42]. The CAVI is derived from stiffness parameter *β*, representing arterial distensibility, using the equation: CAVI = a × (stiffness parameter *β*) + b = a × [2*ρ*/(SBP − DBP) × ln(SBP/DBP) × (haPWV)^2^] + b, where SBP is systolic blood pressure, DBP is diastolic blood pressure, *ρ* is the blood density, and a and b are constants to fit the CAVI values to those of Hasegawa’s PWV [43]. It reflects the stiffness from the ascending aorta to the ankle artery theoretically independently of changes in blood pressure [44]. The CAVI has been demonstrated to have prognostic value for arteriosclerotic diseases, and is increased in patients with cardiovascular disease [45], cerebrovascular disease [46], hypertension [47] and diabetes mellitus [48]. In addition, the CAVI has been shown to be superior to baPWV in predicting coronary artery disease [49,50]. Taken together, these findings imply that haPWV may be a better parameter than baPWV in detecting arterial stiffness.

There were several limitations to this study. First, we did not measure central PWV (cfPWV and hcPWV) or peripheral PWV (faPWV). As a result, we do not know whether central elastic arteries or peripheral muscular arteries were affected by aldosterone first. In addition, the contribution of central and peripheral arteries in the reversal of arterial stiffness is still unclear. Further comprehensive registries may be needed to reflect the time course of aldosterone-induced vascular damage. Second, the number of antihypertensive drugs used was slightly higher and the hypertension duration was longer in the PA group. In addition, the types of medications were not equally distributed between the PA and EH groups. All these factors may have influenced the PWV measurements, which should thus be interpreted with caution. Third, there were 20% PA and 4% EH patients already using spironolactone. This might theoretically lessen the difference between PA and EH patients. The study results did not change even when we only chose patients without spironolactone use in analysis (data not shown).

## 5. Conclusions

In this study, haPWV was superior to baPWV in detecting aldosterone-induced arterial stiffness in PA patients.

## Figures and Tables

**Table 1:** Clinical characteristics of primary aldosteronism and essential hypertension.

	Original Data			Propensity Score Matching of Age and Sex			Propensity Score Matching of Age, Sex, Systolic and Diastolic Blood Pressure		
Patient characteristics	PA (*n* = 1006)	EH (*n* = 983)	*p* value	PA (*n* = 900)	EH (*n* = 900)	*p* value	PA (*n* = 820)	EH (*n* = 820)	*p* value
Sex (Male), *n* (%)	449 (45)	514 (52)	0.001	426 (47)	436 (48)	0.637	380 (46)	390 (48)	0.621
Age, years	54 ± 12	50 ± 15	<0.001	53 ± 12	52 ± 14	0.104	53 ± 12	53 ± 14	0.880
Body height, cm	163 ± 8	164 ± 9	0.001	163 ± 8	163 ± 9	0.781	163 ± 8	163 ± 9	0.760
Body weight, Kg	68 ± 14	69 ± 14	0.144	68 ± 14	68 ± 13	0.252	68 ± 14	68 ± 14	0.834
Body mass index, kg m^−2^	25 ± 4	25 ± 4	0.707	26 ± 4	25 ± 4	0.118	26 ± 4	25 ± 4	0.974
SBP, mmHg	153 ± 21	145 ± 21	<0.001	153 ± 22	145 ± 21	<0.001	149 ± 20	147 ± 20	0.066
DBP, mmHg	91 ± 14	86 ± 13	<0.001	91 ± 14	86 ± 14	<0.001	89 ± 13	88 ± 13	0.097
Serum creatinine level, mg dL^−1^	0.9 ± 0.5	1.0 ± 0.9	0.132	0.9 ± 0.4	1.0 ± 1.0	0.169	0.9 ± 0.5	1.0 ± 1.0	0.091
Serum potassium level, mmol L^−1^	3.7 ± 0.6	4.1 ± 0.4	<0.001	3.7 ± 0.6	4.1 ± 0.4	<0.001	3.7 ± 0.6	4.1 ± 0.4	<0.001
APA, *n* (%)	636 (63)	-	-	575 (64)	-	-	521 (64)		
PAC, ng dL^−1^	42 (29–61)	34 (23–50)	<0.001	42 (29–61)	33 (22–50)	<0.001	42 (30–61)	33 (22–50)	<0.001
PRA, ng mL^−1^ h^−1^	0.3 (0.1–0.6)	1.7 (0.5–4.6)	<0.001	0.3 (0.1–0.6)	1.6 (0.5–4.4)	<0.001	0.3 (0.1–0.6)	1.5 (0.4–4.1)	<0.001
ARR	169 (58–463)	21 (9–63)	<0.001	169 (60–479)	22 (9–67)	<0.001	162 (61–464)	22 (9–71)	<0.001
Log-transformed PAC	1.6 ± 0.3	1.5 ± 0.3	<0.001	1.6 ± 0.3	1.5 ± 0.3	<0.001	1.6 ± 0.3	1.5 ± 0.3	<0.001
Log-transformed PRA	−0.6 ± 0.7	0.1 ± 0.7	<0.001	−0.6 ± 0.7	0.1 ± 0.7	<0.001	−0.6 ± 0.7	0.1 ± 0.7	<0.001
Log-transformed ARR	2.3 ± 0.7	1.4 ± 0.7	<0.001	2.3 ± 0.7	1.4 ± 0.7	<0.001	2.3 ± 0.7	1.5 ± 0.7	<0.001
Number of antihypertensive medication type	2.0 ± 1.3	1.4 ± 1.1	<0.001	2.0 ± 1.3	1.4 ± 1.1	<0.001	1.9 ± 1.3	1.5 ± 1.1	<0.001
Hypertension history, years	7.8 ± 8.1	5.1 ± 6.8	<0.001	7.6 ± 8.0	5.2 ± 6.9	<0.001	7.3 ± 7.7	5.3 ± 7.0	<0.001
Hypertension medication									
ACEI, *n* (%)	18 (2)	17 (2)	0.919	16 (2)	17 (2)	0.861	18 (2)	17 (2)	0.864
ARB, *n* (%)	377 (38)	403 (41)	0.108	345 (38)	376 (42)	0.136	301 (37)	344 (42)	0.030
Alpha-blocker, *n* (%)	206 (21)	112 (11)	<0.001	190 (21)	98 (11)	<0.001	156 (19)	95 (12)	<0.001
Beta-blocker, *n* (%)	353 (35)	202 (21)	<0.001	313 (35)	193 (21)	<0.001	280 (34)	175 (21)	<0.001
CCB, *n* (%)	649 (65)	543 (55)	<0.001	573 (64)	499 (55)	<0.001	531 (65)	456 (56)	<0.001
Vasodilator, *n* (%)	63 (6)	19 (2)	<0.001	54 (6)	19 (2)	<0.001	49 (6)	18 (2)	<0.001
Spironolactone, *n* (%)	201 (20)	36 (4)	<0.001	155 (20)	32 (4)	<0.001	161 (20)	33 (4)	<0.001
Diuretics, *n* (%)	110 (11)	55 (6)	<0.001	80 (10)	48 (6)	0.003	84 (10)	49 (6)	0.002

Abbreviation: PA, primary aldosteronism; EH, essential hypertension; SBP, systolic blood pressure; DBP, diastolic blood pressure; APA, aldosterone-producing adenoma; PAC, plasma aldosterone concentration; PRA, plasma renin activity; ARR, aldosterone-renin ratio; ACEI, angiotensin-converting enzyme inhibitor; ARB, angiotensin receptor blocker; CCB, calcium channel blocker.

**Table 2:** Pulse wave analysis of primary aldosteronism and essential hypertension.

	Original Data			Propensity Score Matching of Age and Sex			Propensity Score Matching of Age, Sex, Systolic and Diastolic Blood Pressure		
Pulse wave analysis	PA (*n* = 1006)	EH (*n* = 983)	*p* value	PA (*n* = 900)	EH (*n* = 900)	*p* value	PA (*n* = 820)	EH (*n* = 820)	*p* value
baPWV (cm/s)	1637 (1452–1868)	1527 (1362–1756)	<0.001	1616 (1440–1844)	1544 (1371–1776)	<0.001	1602 (1434–1816)	1574 (1394–1797)	0.047
haPWV (cm/s)	1103 (1008–1218)	1040 (945–1150)	<0.001	1093 (1003–1208)	1052 (957–1158)	<0.001	1089 (995–1198)	1062 (974–1167)	0.001

Abbreviation: PA, primary aldosteronism; EH, essential hypertension; baPWV brachial-ankle pulse wave velocity; haPWV, heart-ankle pulse wave velocity.

**Table 3:** Area under the ROC curve (AUC) between baPWV and haPWV.

	Original Data			Propensity Score Matching of Age and Sex			Propensity Score Matching of Age, Sex, Systolic and Diastolic Blood Pressure		
	baPWV	haPWV	*p* value	baPWV	haPWV	*p* value	baPWV	haPWV	*p* value
AUC [95% CI]	0.5916 [0.5667–0.6165]	0.6165 [0.5920–0.6411]	0.0001	0.5610 [0.5346–0.5875]	0.5854 [0.5592–0.6116]	0.0001	0.5284 [0.5004–0.5563]	0.5473 [0.5195–0.5751]	0.0046

Abbreviation: AUC, areas under receiver operating characteristic curve; baPWV brachial-ankle pulse wave velocity; haPWV, heart-ankle pulse wave velocity.

## Data Availability

The raw data supporting the conclusion of this manuscript will be made available by the authors, without undue reservation, to any qualified researcher.

## Appendix A

Membership of the Taiwan Primary Aldosteronism Investigation (TAIPAI) Study Group: Che-Hsiung Wu, MD (Chi-Taz Hospital, PI of Committee); Vin-Cent Wu, MD (NTUH, PI of Committee); Yen-Hung Lin, MD (NTUH, PI of Committee); Hung-Wei Chang, MD, PhD (Far Eastern Clinics, PI of Committee); Lian-Yu Lin MD, PhD (NTUH, PI of Committee); Fu-Chang Hu, MS, ScD, (Harvard Statistics, Site Investigator); Kao-Lang Liu, MD (NTUH, PI of Committee); Shuo-Meng Wang, MD (NTUH, PI of Committee); Kuo-How Huang, MD (NTUH, PI of Committee); Yung-Ming Chen, MD (NTUH, PI of Committee); Chin-Chen Chang, MD (NTUH, PI of Committee); Shih-Cheng Liao, MD (NTUH, PI of Committee); Ruoh-Fang Yen, MD, PhD (NTUH, PI of Committee); and Kwan-Dun Wu, MD, PhD (NTUH, Director of Coordinating Center).
